# Dr. Frank W. Whitcomb

**Published:** 1904-08

**Authors:** 


### OBITUARY.

Dr. Frank W. Whitcomb, of Warren, Pa., a graduate of the University of Buffalo, 1882, was drowned in Conewango Creek, near Frewsburg, N. Y., June 30, 1904, aged 45 years.

